# A creeping eruption

**DOI:** 10.1016/j.jdcr.2024.10.025

**Published:** 2024-11-16

**Authors:** Eleonora De Luca, Luca Pellegrino, Giacomo Caldarola

**Affiliations:** a Dermatologia, Dipartimento di Medicina e Chirurgia Traslazionale, Università Cattolica del Sacro Cuore, Rome, Italy; b UOC di Dermatologia, Dipartimento di Scienze Mediche e Chirurgiche, Fondazione Policlinico Universitario A Gemelli - IRCCS, Rome, Italy

**Keywords:** cutaneous pili migrans, foot sole, larva migrans, linear lesion

## Case presentation

A 5-month-old male infant presented with an asymptomatic black lesion on his left sole.

**Fig 1:**
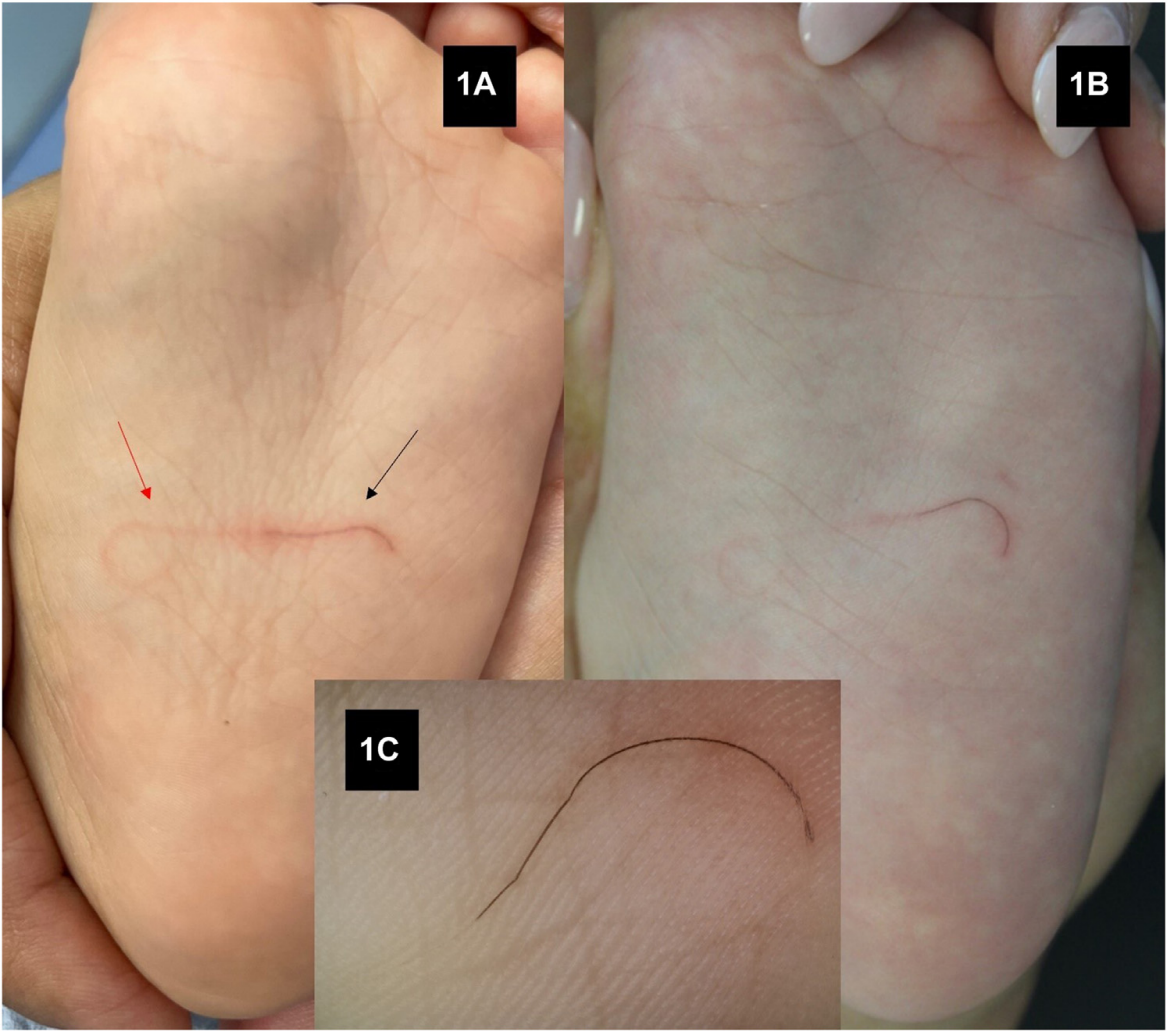

On physical examination, the posterior end of this linear black lesion (black arrow) was contiguous with a curvilinear slightly erythematous lesion (red arrow) of similar length (Fig 1, *A*). The child seemed not pained and his mother referred no previous history of injury in the affected area.

After 4 days, the black segment migrated laterally, assuming a curvilinear shape and leaving an erythematous trail (Fig 1, *B*). Dermoscopy showed a dark line in the superficial skin layer (Fig 1, *C*).

**Question 1: What is the most likely diagnosis?**

A. Scabies
B. Cutaneous pili migrans (CPM)
C. Larva migrans
D. Dermatophyte infection
E. Dermatitis artefacta

**Answers:**

A. Scabies – Incorrect. Scabies causes intense itching and burrows in the skin, but these are typically smaller in length (1-10 mm), skin-colored, and do not present as migrating black lesions.
B. Cutaneous pili migrans (CPM) – Correct. CPM is a rare dermatological condition characterized by the migration of a hair fragment within the superficial layers of the skin, leading to linear erythema. It usually follows an injury and typically involves frictional areas of the skin.^1^ Curettage confirmed CPM, revealing a black hair without follicle.
C. Larva migrans – Incorrect. Cutaneous larva migrans is the main differential diagnosis: this condition involves a tortuous migration pattern with intense itching, differing from CPM’s linear migration and asymptomatic presentation.^2^
D. Dermatophyte infection – Incorrect. Typically associated with scaling and itching, dermatophyte infections do not usually present with the described migration pattern or dermoscopic findings.
E. Dermatitis artefacta – Incorrect. Dermatitis artefacta refers to skin lesions that mimic other conditions but are caused by self-inflicted trauma or behavior, which is unlikely in a 5-month-old male infant.

**Question 2: What is the most likely etiology of cutaneous pili migrans?**

A. Hair fragment migration in the skin
B. Infection by parasitic worms
C. Bacterial skin infection
D. Allergic reaction
E. Fungal infection

**Answers:**

A. Hair fragment migration in the skin – Correct. CPM can result from an ingrown hair burrowing in the dermis or from a foreign body, such as a hair shaft, entering the epidermis due to friction.
B. Infection by parasitic worms – Incorrect. CPM is not caused by parasitic worms, which are associated with conditions like cutaneous larva migrans.
C. Bacterial skin infection – Incorrect. CPM is not due to bacterial infection but rather involves a hair fragment migrating within the skin.
D. Allergic reaction – Incorrect. Allergic reactions cause different types of skin lesions and are not related to CPM.
E. Fungal infection – Incorrect. CPM is not caused by a fungal infection.

**Question 3: What is the recommended treatment approach for cutaneous pili migrans?**

A. Cryotherapy
B. Topical antiparasitic creams
C. Systemic antifungal medication
D. Oral antihistamines
E. Extraction/Curettage

**Answers:**

A. Cryotherapy – Incorrect. Cryotherapy is not used for CPM.
B. Topical antiparasitic creams – Incorrect. Topical antiparasitic creams are not effective for CPM, which is caused by a hair fragment, not a parasite.
C. Systemic antifungal medication – Incorrect. Systemic antifungal medication is not appropriate for CPM, which is not caused by a fungal infection.
D. Oral antihistamines – Incorrect. Oral antihistamines address itching but do not remove the hair fragment responsible for CPM.
E. Extraction/Curettage – Correct. If the hair fragment is protruding from one end, extraction with forceps can be an option. Otherwise, curettage is an effective, painless treatment, as it allows for the removal of the hair fragment by gently removing the overlying layer of skin. Considering CPM in the differential diagnosis when faced with creeping eruptions can prevent unnecessary topical or systemic antiparasitic treatments.

## Conflicts of interest

None disclosed.
